# Examining the time course of post collaborative benefits across word lists and prose passages

**DOI:** 10.3758/s13421-024-01609-5

**Published:** 2024-07-26

**Authors:** Yunfeng Wei, Brooke Z. Charbonneau, Michelle L. Meade, Keith A. Hutchison

**Affiliations:** Montana State University, Department of Psychology, P.O. Box 173440, Bozeman, MT USA

**Keywords:** Collaborative inhibition, Post collaborative benefits, Error correction, Delay

## Abstract

In the current study, we investigated how long the effects of one single collaboration session continue to influence individual memory. Participants learned categorized word lists and prose passages individually, and then they were instructed to recall learned materials either collaboratively or individually. Following initial recall, participants completed an individual recall test after a delay of 5 min, 48 h, or 1 week. On the initial recall test, we found that collaboration reduced recall of correct items on both word lists and prose passages (collaborative inhibition), and that collaboration reduced false recall on both word lists and prose passages (error correction). However, on the subsequent individual memory test after a delay, the pattern of post collaborative effects differed across veridical and false recall. For both word lists and prose passages, post collaborative benefits on correct recall lasted 1 week. However, there were no lasting effects of error correction on subsequent false recall. These results suggest that the time course of post collaborative benefits can be long lasting, but they are selective to veridical recall. The results are explained by theories of reexposure and error correction.

We often recall our memories with other people, such as an interesting childhood story, a familiar melody, or a specific moment in life, but collaborative recall may induce two phenomena—collaborative inhibition and post collaborative benefits (Meade et al., 2018). Collaborative inhibition refers to group memory performance being inferior to pooled non-redundant memory performance of the same number of individuals working separately (e.g., Weldon & Bellinger, 1997; see Rajaram, 2018, for review). Post collaborative benefits refer to the enduring effects of collaboration; on subsequent individual tests, those individuals who previously collaborated remember more than those who previously recalled alone (e.g., Abel & Bäuml, 2017; Basden et al., 2000; see Rajaram, 2018, for review). The purpose of the current study is to explore the time course of collaborative inhibition and post collaborative benefits. That is, how soon after collaboration do post collaborative benefits emerge, and how long do they last?

## Collaborative inhibition and post collaborative benefits

Collaborative inhibition is measured by comparing collaborative group performance and nominal group performance—non-redundant pooled performance of individuals working separately. For example, individuals learned items A, B, C, D, E, F, and G, and two of them try to recall the learned items separately. The first one recalls A, C, and D individually, and the second one recalls A, B, D, and F individually. Then their nominal group performance is A, B, C, D, F. Collaborative inhibition refers to the findings that nominal group performance is better than collaborative group performance in general (see Meade et al., 2018, for a review; cf. Meade et al., 2009). Collaborative groups recall more than individuals working alone, but less than individuals pooled together into a nominal group.

Multiple mechanisms underlie collaborative inhibition. Basden et al. (1997) claimed that collaborative inhibition can be explained by the retrieval strategy disruption theory. According to retrieval strategy disruption, when individuals learn some materials, they form an idiosyncratic organization of those materials, and they can later retrieve learned information based on their organization. However, when individuals recall learned materials with others, their organization is disrupted by the output of other people, leading to collaborative inhibition. More recently, there is growing evidence that collaborative inhibition is multiply determined. Specifically, the magnitude of collaborative inhibition is influenced by retrieval inhibition (unrecalled items are inhibited during collaboration). Barber et al. (2015) demonstrated retrieval inhibition by showing long-lasting effects of collaborative deficits on subsequent tests. Collaborative inhibition is also influenced by attentional control; Hood et al. (2023) showed that collaborative inhibition was greater for individuals lower in working memory capacity and suggested that these individuals likely have difficulty recalling items when faced with the distraction of a partner recalling items at the same time. Finally, collaborative inhibition is influenced by collaborative process variables, or how groups coordinate information and acknowledge each other’s contributions (Meade, 2013; Meade et al., 2009). Meade and Gigone (2011) demonstrated that collaborative inhibition is larger when groups do not acknowledge and elaborate on each other’s contributions, most likely because the unacknowledged items are less likely to be incorporated into the group’s recall (cf. Ekeocha & Brennan, 2008). Together, these processes explain why collaboration is disruptive to individual memory performance.

In spite of the initial negative disruptive effects of collaboration on memory, collaboration may result in benefits for later individual recall (see Marion & Thorley, 2016). Once the disruptive effects of one’s partner are removed, there is an opportunity for individuals to draw upon their partners’ contributions to benefit their own subsequent memory (cf. Congleton & Rajaram, 2011). According to Rajaram and Periera-Pasarin (2010), the primary mechanism underlying post collaborative benefits is reexposure; recalling learned materials with others provides individuals with a second studying opportunity to be exposed to the information (Blumen & Rajaram, 2008; Blumen & Stern, 2011; Weldon & Bellinger, 1997; for reviews, see Rajaram, 2018; Rajaram & Pereira-Pasarin, 2010). Cross cueing is also possible (the outputs of group members can cue each other to help them recall information they are not able to recall without a cue); however, there is very little evidence supporting cross cueing (see Meudell et al., 1992, 1995).

Importantly, although much evidence exists that collaboration produces post collaborative benefits, this is not always the case. Specifically, several studies have found no difference in subsequent recall following collaboration (e.g., Barber et al., 2015; Blumen & Rajaram, 2008; Finlay et al., 2000; Meade & Roediger, 2009; Wright & Klumpp, 2004), whereas others have found only marginal benefits (e.g., Whillock et al., 2020). Still others suggest that the magnitude of post collaborative benefits is tied to the magnitude of collaborative inhibition, such that smaller collaborative inhibition effects are more likely to produce post collaborative benefits (e.g., Abel & Bäuml, 2017; Congleton & Rajaram, 2011; Hood et al., 2023). Given the mixed findings, it is important to further examine these effects. The present experiment adds to the current understanding of post collaborative benefits by systematically examining the time course of post collaborative benefits across different stimuli (word lists and prose passages).

## Time course of post collaborative benefits

Examining the time course of post collaborative benefits is critical for understanding any lasting impact of collaboration on individual memory. Previous research has examined the effects of delay on collaborative inhibition and post collaborative benefits; however, the delay has typically been manipulated between study and initial collaboration, rather than between initial collaboration and subsequent individual test, and the subsequent individual test occurs immediately, or very soon after, collaboration. For example, Takahashi and Saito (2004) asked participants to collaboratively recall information immediately after learning stimuli (Experiment 1) or after a 1-week delay (Experiment 2). Immediately after the collaboration, participants were instructed to complete a final individual recall. The authors found that collaborative inhibition was removed when individuals collaborated after a long delay, and post collaborative benefits were detected on the immediately subsequent individual test. Congleton and Rajaram (2011) examined how a short (7 min) and long (120 min) delay between study and collaboration influenced collaborative inhibition and post collaborative benefits. Their findings suggested that a longer delay between learning and collaboration decreases collaborative inhibition and increases post collaborative benefits. Also, Abel and Bäuml (2017) compared the effect of a short delay (5 min) and a long delay (24 h) between learning and collaboration, and they claimed that a long delay can effectively attenuate collaborative inhibition and improve post collaborative benefits on an immediate subsequent individual test. Finally, the benefits of a delay between study and collaboration generalize to recognition memory performance (Rajaram & Pereira-Pasarin, 2007). Together, these studies demonstrate that a delay before collaboration can influence both collaborative inhibition and post collaborative benefits, presumably because retrieval organization declines over time and so there is relatively less to disrupt and potentially greater gains from reexposure (cf. Congleton & Rajaram, 2011). However, they cannot answer questions about how long the effects of collaboration continue to influence memory.

In the current study, we are interested in the role of delay following, rather than before, collaboration. That is, how long do the effects of collaboration continue to influence individual memory? To our knowledge, just two studies have examined delay between collaboration and final individual recall, and they found somewhat inconsistent results. Blumen and Stern (2011) asked participants to complete three immediate successive recall tasks of unrelated word lists (collaborative, collaborative, individual, or individual, individual, individual) and then return 1 week later for a final individual recall test. They found higher recall on the final test following repeated collaboration, demonstrating that post collaborative benefits can last up to 1 week. However, these results are confounded by the multiple tests intervening between collaboration and the 1-week delayed recall. In contrast, Yaron-Antar and Nachson (2006) found no evidence of long-lasting collaborative benefits regarding the number of accurate details recalled. However, when the number of accurate items was considered in relation to the smaller number of inaccurate items produced in collaborative groups, prior collaboration led to proportionally higher accuracy rates. They asked participants to complete short-answer and multiple-choice tests about the assassination of Israel’s prime minister individually or collaboratively, and to return 1 week later to remember again either individually or collaboratively. Participants in the individual–individual condition produced numbers of accurate details (but fewer inaccurate details, resulting in a higher rate of accurate details) that were similar to participants in the collaborative–individual condition, suggesting that any lasting effects of collaboration were selective to this measure. However, this study involved recall of a highly important public event, and so there are potential confounds with extra-experimental conversations and news stories. In the current study, we rely on word lists and prose passages and include just one collaborative–individual recall test prior to subsequent recall so as to be consistent with the paradigm used in the majority of collaborative inhibition studies.

## Does collaboration produce a “desirable difficulty”?

Our interest in delay between initial collaboration and final test is also based on the testing effect literature (see Roediger & Karpicke, 2006a, for review). The testing effect demonstrates that the benefits of testing over repeated study typically appear only after a delay. Specifically, individuals who restudy learned materials perform better than those who retrieve learned materials immediately after a restudying/retrieving session. However, individuals who retrieve learned materials perform better than those restudying materials after a long delay. That is, even though testing/retrieval practice impairs short-term performance, it improves long-term performance/learning. One explanation is that testing is a “desirable difficulty” such that the increased effort, associations, and practice during testing promote long term learning (e.g., Bjork, 1994). To connect testing effects to the collaborative inhibition literature, one possibility is that the initial disruptive aspects of collaboration that induce collaborative inhibition produce a desirable difficulty. If collaboration creates a desirable difficulty during initial recall, the increased effort and potential for new connections and cueing during collaboration may convert to advantages over time. More specifically, it is possible that trying to maintain to-be-recalled items in the face of distraction strengthens them and/or the interference during collaboration causes more effortful retrieval for the items produced. Importantly, however, the time course is again critical. Desirable difficulty benefits typically appear only on delayed tests (e.g., 48-h, 1-week; Roediger & Karpicke, 2006b), whereas post collaborative benefits typically appear on immediate and short delay tests (e.g., 0–10 min; e.g., Thorley & Dewhurst, 2007; Weldon & Bellinger, 1997).

For this reason, in the current study we directly compare the magnitude of post collaborative benefits following a 5-min delay, a 48-h delay, and a 1-week delay. Based on previous research showing post collaborative benefits, at 5-min delay, we predict either a collaborative advantage or no difference between prior collaborative and prior individual recall. At a 48-h delay, we predict significant post collaborative benefits; at 1-week, we predict significant post collaborative benefits. However, if collaboration is a desirable difficulty, we predict worse performance following collaboration on the 5-min test, but better performance on the 48-h and 1-week delayed tests. That is, both reexposure and desirable difficulties predict post collaborative benefits after 48-h and 1-week delays. However, there are different predictions for the 5-min delay condition: reexposure predicts post collaborative benefits after a 5-min delay, whereas desirable difficulties predict no post collaborative benefits after a 5-min delay.

## Generalizability of post collaborative benefits across word lists and prose passages

The current study examines the time course of post collaborative benefits following collaborative recall of word lists and prose passages. Whereas collaborative inhibition has been obtained across a range of materials including word lists (e.g., Henkel & Rajaram, 2011), short stories (e.g., Weldon & Bellinger, 1997), socially relevant information (e.g., Kelley et al., 2012), and emotional materials (e.g., Wessel et al., 2015), much less is known about possible similarities and differences in post collaborative benefits that result from prior collaboration of different materials. Marion and Thorley (2016) concluded there is evidence for post collaborative benefits following prior collaboration on immediate tests (0–10 minute delay), and further, that these post collaborative benefits were least likely for items high with interitem association. However, again, little is known about post collaborative benefits on delayed tests and, to our knowledge, no studies have directly compared materials on delayed tests. Further, work on the testing effect suggests that materials with higher associative overlap benefit more from testing because individuals can continue to process, synthesize, and associate the concepts over time (e.g., Szpunar et al., 2007). As such, in the current experiment we chose categorized word lists and prose passages. Categorized words lists are composed of exemplars, whereas prose passages are composed of idea units. The inclusion of both materials tests the generalizability of any findings across multiple stimuli. In the current study, we predict similar patterns of post collaborative effects for both word lists and prose passages over time, but it is also possible that prose passages could have larger and longer lasting effects because they include multiple idea units associated with each concept, and if a concept is cued by other group members, more related idea units in the prose passages are cued.

In addition to examining correct recall, using word lists and prose passages allows us to code for errors. One advantage of collaboration is that group members can correct each other’s errors (e.g., Ross et al., 2008), leading collaborative groups to recall fewer errors than nominal groups. However, relatively little is known about how long such error correction lasts. Some evidence suggests reduced errors during collaboration also results in reduced errors on subsequent individual tests (e.g., Rossi-Arnaud et al., 2020). However, collaboration can also increase errors on subsequent tests, when collaborators discuss errors without correcting them (e.g., Meade & Roediger, 2009; cf. Meade & Roediger, 2002; Roediger et al., 2001). Further, little is known regarding differences in the time course of different types of false memory errors (e.g., associative, categorical, schematic; cf. Roediger, 1996). In the current experiment, we directly compare the lasting effects of collaboration on errors produced across prose passages and word lists. For collaborative recall of these materials, we rely on free-for-all collaboration (participants freely interact with each other and recall is unstructured) because free-for-all collaboration allows more opportunities for discussion and error correction relative to turn-taking collaboration where participants take turns to recall items (e.g., Saraiva & Garrido, 2024).  Given that we are using free-for-all collaboration, rather than turn-taking, we predict collaborative error correction on the initial test (Thorley & Dewhurst, 2007). Further, we predict error correction will persist on subsequent tests. Finally, we predict similar patterns for both word lists and prose passages, but perhaps larger effects for word lists because error correction is more easily tied to single items than to idea units (in which errors may be related to details and/or main concepts of each idea unit).

## The present study

In this study, we aimed to answer two questions: (1) how post collaborative benefits change over time; (2) whether the time course of collaborative inhibition and post collaborative benefits can generalize across materials. For both questions, we examined both accurate and false recall. Of interest is when/how collaboration continues to influence individual recall after a delay.

## Method

### Participants

Marion and Thorley (2016) reported the effect size for collaborative inhibition as Cohen’s *d* = 0.56 and the effect size for post collaborative benefits as Cohen’s* d* = 0.59. We felt it was necessary to replicate collaborative inhibition before examining any lasting effects of collaboration. Therefore, we used the more conservative effect size of 0.56 in our power analysis. Specifically, we conducted a power analysis using G*Power using the effect size of 0.56 reported by Marion and Thorley (2016). However, G*Power uses Cohen’s *f*, rather than Cohen’s *d*. Cohen’s *f* is *d*/2 (https://www.escal.site/) so we used an effect size of 0.28. Assuming an effect size of 0.28 and six groups, the total required sample size to achieve a power of 0.80 is 127 participants. Because dyads are required to measure collaborative inhibition on Recall Test 1 (pairs of individuals are entered as a single collaborative group or a single nominal group), we doubled this number to 254 participants total. In order to equate the number of participants across the six conditions, we chose 252 total participants, or 21 dyads per condition. It is worth noting that the number of participants in the current study did not completely meet the specifications for examining collaborative inhibition, but this number exceeded the required number of participants for detecting post collaborative benefits. Moreover, Whillock et al. (2020) had 12 dyads per conditions (also six between-subject groups), so we are confident that 21 dyads per condition is sufficient power.

We recruited 305 undergraduate students from the Montana State University SONA subject pool and randomly assigned them into different conditions. Students participated in exchange for course credits. Fifty-three students were excluded from data analysis primarily because they did not return for their delayed testing session: 15 students in the individual conditions and 14 students in the collaborative conditions. Fourteen additional participants from the collaborative condition were removed because their partner did not return (note that this exclusion criterion for participants whose partner did not return for Recall 2 was not stated in preregistration, but we later realized it was necessary to ensure that the same participants were included in the Recall 1 analyses and Recall 2 analyses). Finally, eight students were excluded from data analysis because of experimenter error (not labelling the packets or presenting the recall sheets out of order). No students were excluded for failing to follow the instructions or complete the tasks. Thus, 252 participants (21 dyads in each condition) were included in final analyses (mean age = 19.59 years, 33.7% males, 64.3% females, 64.7% first-year students, 93.6% non-Hispanic/Latino, and 91.7% Caucasian). All decisions to exclude participants occurred prior to entering or analyzing the data and were based entirely on notes in the subject log.

### Design

This study was a 2 (collaboration: individual or collaborative) × 3 (delay: 5 min or 48 h, or 1 week) between-subject design. The dependent variables were participants’ correct and false recall of word lists and prose passages. Word list and prose passage recall was measured immediately after learning materials, either in a group or individually, and on a final individual recall test after a delay.

### Materials

Materials included categorized word lists from Meade and Roediger (2006). These lists were created by selecting the top 22 exemplars of each category reported in the Battig and Montague (1969) category norms. The top five most typical exemplars were removed from the list and designated as false items. The next 17 items were designated as study items. Each list contained 17 studied items.

The prose passages were from Roediger and Karpicke (2006a). These passages (“The Sun” and “Sea Otters”) were selected from practice prose passages for the *Test of English as a Foreign Language* (TOEFL; Rogers, 2001). Each passage was a short paragraph that contained 30 idea units. We also used the scoring matrix provided by Karpicke and Roediger.

### Procedure

This study was preregistered at Aspredicted.org (https://aspredicted.org/blind.php?x=2eb5ye) and was approved by the Institutional Review Board (IRB) at Montana State University (MM0309620-EX). All participants signed consent forms and were then randomly assigned to an experimental condition.

#### Session 1: Study-Test 1

Participants were presented with four word lists and instructed to remember them for later memory tests. Each word list contained 17 items, so participants saw 68 items total. Items were blocked by category and the experimenter verbally labelled each category as it began. For example, the experimenter said, “The first list is kitchen utensils,” when participants were about to see words in the kitchen utensils category presented on the computer screen. Each word was presented for 2 s (with a 1-s interstimulus interval). Participants were next asked to complete a filler task. The filler task consisted of double-digit multiplication problems, and participants were asked to solve as many as possible in 2 min. Next, participants were instructed to recall the list either individually or in collaboration with another participant who was their collaborative partner throughout the entire procedure. Participants in the collaborative condition were given “free-for-all” instructions, meaning that they were free to collaborate however they wished. One participant was randomly assigned to be the recorder to write down the recall (we do not expect memory to differ for the recorder given that there is evidence that verbal and written recall are similar; e.g., Gardiner et al., 1977). Participants in both the collaborative and individual conditions were instructed not to guess. Recall was cued by category. Participants were verbally cued on which category to recall and received a piece of paper with a category name written at the top left of the answer sheet. They completed recall of one category before moving to the next. The order of categories at test matched the order at study. Participants were allowed 2 min to recall each list.

#### Session 1: Study-Test 2

Immediately after finishing list study and recall, participants completed a similar process for the prose passages. Specifically, participants were presented with “The Sun” passage and asked to read and remember it. They were again informed that their memory for the passage would be tested later. The experimenter verbally labelled the passage (e.g., “The Sun”), and participants had as much time as necessary to read through the passage. Participants then repeated this process for the second passage, “Sea Otters.” Again, the experimenter verbally labelled the passage, and again participants had as much time as necessary to read through the passage. Self-paced reading instructions are consistent with previous research (e.g., Bergman & Roediger, 1999) and are necessary to ensure that each participant encodes the stories. When participants finished reading each passage, they were instructed to turn over the sheet of paper. The experimenter recorded the time spent reading. On average, participants spent 107.81 s (*SD* = 74.66) and 110.89 s (*SD* = 47.60) reading “The Sun” and “Sea Otters,” respectively. To ensure that reading time was matched across experimental conditions, we ran separate analyses of variance (ANOVAs) on reading time for “The Sun” passage and the “Sea Otters” passage. For “The Sun,” there was no main effect of collaboration on reading time, *F*(1, 152) = 2.25, *p* = 0.14, *MSE* = 5,549.36, η^2^ = 0.02, and there was also no main effect of delay, *F*(2, 152) = 1.13, *p* = 0.33, *MSE* = 5,549.36, η^2^ = 0.02. Also, there was no significant interaction between collaboration and delay, *F*(2, 152) = 0.43, *p* = 0.65, *MSE* = 5,549.36, η^2^ = 0.01. For “Sea Otters,” there was no main effect of collaboration, *F*(1, 151) = 1.89, *p* = 0.17, *MSE* = 2,260.76, η^2^ = 0.01, or delay on their reading time *F*(2, 151) = 1.50, *p* = 0.23, *MSE* = 2,260.76, η^2^ = 0.02. Again, there was no significant interaction between collaboration and delay, *F*(2, 151) = 0.34, *p* = 0.71, *MSE* = 2,260.76, η^2^ < 0.01.

After participants read both passages, they were provided with a mathematical filler task and asked to complete as many multiplication problems as possible in 2 min. Next, participants were instructed to remember the prose passages on their own or in collaboration with a fellow participant. Note that individual and collaborative group conditions were held constant across the experiment so that those who completed list recall individually also completed prose recall individually, and those who completed list recall collaboratively also completed prose recall collaboratively. Again, collaborative groups were instructed to recall freely, and one was assigned recorder. Passages were presented in the same order as during study, and the experimenter provided the same verbal labels of each passage as during study. Participants had 4 min to recall as much as possible from each passage. Participants took about 30–45 min to complete Session 1.

#### Session 2

Session 2 occurred 5 min, 48 h, or 1 week after Session 1. Those participants in the 5-min condition remained in the lab and completed a mathematical filler task for 5 min. Participants in the 48-h condition and participants in the 1-week condition left the lab and returned after their appropriate delay. After the 5-min, 48-h, or 1-week delay, all participants completed an individual recall test. Participants were first asked to remember the word lists. As in Session 1, word lists were presented in the same order as study, and they were verbally labelled with category names. Participants had 2 min to recall each list. Next, participants were asked to remember the prose passages. As in Session 1, the prose passages were verbally labelled and presented in the same order as study. Participants had 4 min to complete recall of each prose passage. Finally, participants completed a post experimental demographics questionnaire, were debriefed, thanked, and awarded class credit for their participation. Participants took about 15–20 min to complete Session 2.

## Results

### Recall 1

#### Correct recall

Table 1 presents the mean proportion of list items recalled and the mean proportion of idea units recalled on the initial test as a function of collaboration and future delay. Note that there was no delay present for the initial test (all groups completed the initial recalls immediately after study), so the delay corresponds to future delay. We computed the nominal group performance by pooling the non-redundant items recalled by two participants in the individual condition. For example, we pooled the performance of Participants 1 and 2 in the individual condition as the nominal group performance of Group 1. Then, we could compute collaborative inhibition by comparing nominal group recall to collaborative group recall (the combined output of two individuals who recalled together). Looking first at list recall, a 2 (collaboration: nominal or collaborative) × 3 (future delay: 5 min, 48 h, or 1 week) between-subjects ANOVA, computed on the proportion of list items recalled, revealed a significant main effect of collaboration, *F*(1, 120) = 18.76, *MSE* = 0.01, *p* < 0.001, η^2^ = 0.14. Replicating the collaborative inhibition effect, those who recalled in a collaborative group (*M* = 0.52) recalled less than the pooled nominal groups (*M* = 0.60). Also important is that no other main effects of delay, *F*(2, 120) = 0.60, *MSE* = 0.01, *p* = 0.55, η^2^ = 0.14 or interactions were significant, *F*(2, 120) = 0.31, *MSE* = 0.01, *p* = 0.74, η^2^ = 0.01. This means that recall performance and the magnitude of the collaborative inhibition effect did not differ across future delay conditions. This matched performance on initial recall is important because there are no scaling effects, and we can more easily interpret any changes on the subsequent delayed tests (see Meade & Roediger, 2006, 2009; Meade et al., 2012, for discussion).

**Table 1:** Mean proportion (standard deviation) of correct list items and correct prose recalled on Test 1 as a function of retrieval condition and future delay (*N* = 252)

	5 min	48 h	1 week
Word lists			
Pooled individual	0.60 (0.10)	0.60 (0.09)	0.60 (0.09)
Collaborative	0.54 (0.09)	0.52 (0.09)	0.50 (0.12)
Prose passages			
Pooled individual	0.53 (0.08)	0.53 (0.09)	0.54 (0.09)
Collaborative	0.43 (0.13)	0.47 (0.06)	0.43 (0.10)

Turning next to prose passage recall, we scored the prose passages according to the scoring manual provided by Roediger and Karpicke (2006a). Two raters double scored 24.6% of the passages. We then computed Kappa on the two sets of coding. The two coders then discussed their disagreements for Kappas less than 60 and came to agreement in order to reach 60% agreement (Landis & Koch, 1977; McHugh, 2012), and then one coder finished the data set. A 2 (collaboration: nominal or collaborative) × 3 (future delay: 5 min, 48 h, or 1 week) between-subjects ANOVA was computed on prose passage recall. The ANOVA revealed significant collaborative inhibition for the prose passages, *F*(1, 120) = 26.78, *MSE* = 0.01, *p* < 0.001, η^2^ = 0.18. Again, participants in the pooled nominal groups (*M* = 0.53) recalled more information from the prose passages than participants in the collaborative groups (*M* = 0.44). Importantly, no other main effects of delay, *F*(2, 120) = 0.54, *MSE* = 0.01, *p* = 0.58, η^2^ = 0.01 or interactions were significant, *F*(2, 120) = 0.89, *MSE* = 0.01, *p* = 0.42, η^2^ = 0.02. As with list recall, initial recall of the prose passages and the magnitude of the collaborative inhibition effect was matched across conditions, which benefits our ability to examine differences on subsequent tests (cf. Meade & Roediger, 2009).

#### False recall

Table 2 presents the mean proportion of nonpresented word list exemplars recalled by participants as a function of collaborative or nominal recall and future delay. The top five most typical exemplars from each categorized list were not presented during study, and the error rates reported in Table 2 represent the mean proportion of those items recalled (cf. Meade & Roediger, 2006, 2009). A 2 (collaboration: collaborative or nominal recall) × 3 (future delay: 5 min, 48 h, or 1 week) between-subjects ANOVA, computed on false recall, demonstrated error correction in the collaborative groups, *F* (1, 120) = 38.65, *MSE* = 0.03, *p* < 0.001, η^2^ = 0.24. Consistent with previous research demonstrating collaborative inhibition for errors, or error correction in collaborative groups (e.g., Ross et al., 2008), collaborative groups in the current experiment recalled fewer errors (*M* = 0.15) than nominal groups (*M* = 0.35). No other main effects of delay, *F*(2, 120) = 0.18, *MSE* = 0.03, *p* = 0.84, η^2^ < 0.01, or interactions were significant, *F*(2, 120) = 0.06, *MSE* = 0.03, *p* = 0.94, η^2^ < 0.01, suggesting the magnitude of false recall and error correction were matched across conditions on Recall Test 1.

**Table 2:** Mean proportion (standard deviation) of false list items recalled on Test 1 as a function of retrieval condition and future delay (*N* = 252)

	5 min	48 h	1 week
Word lists			
Pooled individual	0.36 (0.23)	0.33 (0.19)	0.36 (0.23)
Collaborative	0.16 (0.14)	0.14 (0.11)	0.14 (0.16)

Table 3 presents the mean number of extra list intrusions recalled for the prose passages. The mean number of intrusions is reported (rather than the proportion of nonpresented exemplars) because the prose passages were not designed to exclude specific information. Measuring false recall on prose passages as the number of extra list intrusions is consistent with previous research (e.g., Roediger & Karpicke, 2006a, 2006b). Specifically, we operationally defined false recall as any idea unit that was inconsistent with the idea units presented (e.g., sea otters eat kelp; the passage stated they protect kelp and sleep on kelp) or idea units that were not presented at all in the scenarios (e.g., sea otters hold hands; the passage stated nothing about this). Overall error rates were low for the passages, so data reported collapse across types of errors. These scoring criteria are consistent with scoring false recall for other prose passage (e.g., Bergman & Roediger, 1999); note that Karpicke and Roediger did not report errors or include a rubric for scoring errors, because this was not relevant to their research questions.

**Table 3:** Mean number (standard deviation) of extralist intrusions recalled on prose Test 1 as a function of retrieval condition and future delay (*N* = 252)

	5 min	48 h	1 week
Prose passages			
Pooled individual	2.33 (3.12)	2.14 (1.80)	2.86 (1.77)
Collaborative	1.43 (1.17)	1.10 (1.51)	1.00 (0.89)

A separate 2 (collaboration: collaborative or nominal recall) × 3 (future delay: 5 min, 48 h, or 1 week) between-subjects ANOVA, computed on the mean number of extra list intrusions on prose passages, revealed that there was a significant main effect of collaboration, *F*(1, 120) = 38.65, *MSE* = 3.42, *p* < 0.001, η^2^ = 0.24, indicating that collaboration can effectively decrease false recall. Specifically, participants in collaborative groups recalled fewer intrusions (*M* = 1.17) than participants in pooled nominal groups (*M* = 2.44). Importantly, there were no other main effects of delay, *F*(2, 120) = 0.34, *MSE* = 3.42, *p* = 0.71, η^2^ = 0.01, or interaction, *F*(2, 120) = 0.81, *MSE* = 3.42, *p* = 0.45, η^2^ = 0.01, suggesting that error correction was matched across groups.

### Recall 2

#### Correct recall

The mean proportions of list recall and prose passage recall on the subsequent individual test are presented in Table 4. All participants completed Recall 2 individually, so the table refers to prior collaborative and prior individual recall to indicate participant condition on Test 1. A 2 (prior collaboration: prior individual or prior collaborative) × 3 (delay: 5 min, 48 h, or 1 week) between-subjects ANOVA was first computed on the proportion of list items recalled. The ANOVA revealed a main effect of prior collaboration, *F*(1, 246) = 7.95, *MSE* = 0.01, *p* < 0.005, η^2^ = 0.03, indicating significant post collaborative benefits for those who previously collaborated (*M* = 0.37) relative to those who previously recalled individually (*M* = 0.34). There was also a main effect of delay, *F*(2, 246) = 39.93, *MSE* = 0.01, *p* < 0.001, η^2^ = 0.25. Recall performance was worse with longer delays (*M* = 0.43 after 5 min, *M* = 0.35 after 48 h, and *M* = 0.29 after 1 week). Then, we conducted follow-up* t* tests to compare the performance of two groups, and if the assumption of equal variances was violated, we reported corrected degrees of freedom and *t* values. Post hoc *t* tests showed that participants in 5 min delay condition performed better than those in the 48-h, *t* (166) = 4.58, *SEM* = 0.08*, p* < 0.001, Cohen’s *d* = 0.70, and 1-week conditions, *t* (151.72) = 8.87, *SEM* = 0.02, *p* < 0.001, Cohen’s *d* = 0.1.37, and participants in the 48-h condition also recalled more correct items than participants in the 1-week condition, *t*(166) = 4.20, *SEM* = 0.01, *p* < 0.001, Cohen’s *d* = 0.65. There was no interaction between prior collaboration and delay, *F*(2, 246) = 0.41, *MSE* = 0.01, *p* = 0.67, η^2^ < 0.01, suggesting that delay reduced recall to the same extent regardless of prior collaborative recall condition. We ran a Bayesian ANOVA in JASP (Version 0.18.3; JASP Team, 2024) for the interaction of prior collaboration and delay. Specifically, we compared the model containing the interaction with the null interaction model that contained both main effects but no interaction. We did not include Bayesian analyses in the preregistration, but we ran this analysis because the Bayesian output provides more details about how the data support different models. JASP provides a default prior of 0.50 when comparing the model with interaction and the model with only main effects, suggesting that both models are considered equally likely. The resulting BF_10_ represents a ratio of how likely the model containing the interaction is over the null model. According to the classification scheme from Lee and Wagenmakers (2013; adjusted from Jeffreys, 1961), a BF_10_ of 10–30 = strong evidence, 3–10 = moderate evidence, 1–3 = anecdotal (weak) evidence, and 1 = no evidence. (Note that values < 1 equal evidence for the null, such that 0.33 and 0.10 equal moderate and strong evidence for the null hypothesis, respectively.) For the interaction effect, we found a BF_10_ = 0.10, which means that, given the data, the null interaction model is 10 times more likely than the model containing the interaction, indicating moderate-to-strong support for the null interaction (van Doorn et al., 2021).

**Table 4:** Mean proportion (standard deviation) of correct list items and correct prose recalled on Test 2 as a function of prior retrieval condition and delay (*N* = 252)

	5 min	48 h	1 week
Word lists			
Prior individual	0.40 (0.11)	0.33 (0.10)	0.27 (0.08)
Prior collaborative	0.45 (0.12)	0.36 (0.10)	0.30 (0.09)
Prose passages			
Prior individual	0.35 (0.11)	0.31 (0.09)	0.25 (0.09)
Prior collaborative	0.41 (0.13)	0.39 (0.11)	0.27 (0.10)

A separate 2 (prior collaboration: prior individual or prior collaborative) × 3 (delay: 5 min, 48 h, or 1 week) between-subjects ANOVA was computed on the proportion of idea units recalled from the prose passages. Consistent with the results reported for word lists, participants showed post collaborative benefits, *F*(2, 246) = 14.64, *MSE* = 0.01, *p* < 0.05, η^2^ = 0.06 such that participants recalled more following collaboration (*M* = 0.36) than following individual recall (*M* = 0.30). There was also a main effect of delay, *F*(2, 246) = 27.29, *MSE* = 0.01, *p* < 0.05, η^2^ = 0.18, with recall decreasing with delay (*M* = 0.38 at 5 min, *M* = 0.35 at 48 h, and *M* = 0.26 at 1 week). Post hoc *t* tests demonstrated that there was no significant difference between 5-min and 48-h delay, *t*(166) = 1.70, *SEM* = 0.03, *p* = 0.09, Cohen’s *d* = 0.26, but participants in the 1-week condition recalled fewer idea units than those in the 5-min condition, *t*(156.88) = 6.86, *SEM* = 0.12, *p* < 0.001, Cohen’s *d* = 1.06, and the 48-h condition, *t*(166) = 5.47, *SEM* = 0.09, *p* < 0.001, Cohen’s *d* = 0.85. Finally, the interaction was again not significant, *F*(2, 246) = 2.08, *MSE* = 0.01, *p* = 0.12, η^2^ = 0.02. Bayes factors BF_10_ = 0.41 when comparing the interaction effect model with the null model (main effects included), and it suggests that there is weak evidence supporting the null (van Doorn et al., 2021).

#### False recall

The mean proportion of incorrect list items recalled on List 2 are presented in Table 5. A 2 (prior collaboration: prior individual or prior collaborative) × 3 (delay: 5 min, 48 h, or 1 week) between-subjects ANOVA was computed on list errors. There was a marginal main effect of delay on error rates that appears to be driven by the 1-week condition (*M* = 0.20 at 5 min, *M* = 0.19 at 48 h, *M* = 0.25 at 1 week), *F*(2, 246) = 2.76, *MSE* = 0.04, *p* = 0.07, η^2^ = 0.02. When this pattern is considered in relation to participants’ decreased veridical recall with delay, overall participants were less accurate across time (cf. Bergman & Roediger, 1999). Importantly, the ANOVA revealed no main effect of collaboration, *F*(1, 246) = 0.99, *MSE* = 0.04, *p* = 0.32, η^2^ < 0.01. Specifically, participants who previously collaborated (*M* = 0.20) recalled similar proportions of false items as participants who previously recalled alone (*M* = 0.19). The error correction evident on Test 1 was short lived and did not persist on Test 2, a finding consistent with previous research showing no lasting effects of error correction on categorized lists (e.g., Hood et al., 2023; Meade & Roediger, 2009; Whillock et al., 2020). Finally, the interaction between collaboration and delay was not significant, *F*(2, 246) = 1.34, *MSE* = 0.04, *p* = 0.26, η^2^ < 0.01. Furthermore, A Bayesian analysis showed the BF_10_ = 0.21, suggesting that the null model (including main effects) is 5 times more likely than the interaction effect model, providing moderate evidence for the null hypothesis.

**Table 5:** Mean proportion (standard deviation) of false list items recalled on Test 2 as a function of prior retrieval condition and delay (*N* = 252)

	5 min	48 h	1 week
Word lists			
Prior individual	0.23 (0.23)	0.21 (0.18)	0.24 (0.22)
Prior collaborative	0.17 (0.17)	0.16 (0.12)	0.27 (0.19)

For prose errors, a separate 2 (prior collaboration: prior collaborative or prior individual recall) × 3 (delay: 5 min, 48 h, or 1 week) between-subjects ANOVA computed on the mean number of extra list intrusions on prose passages revealed no main effect of collaboration, *F*(1, 246) = 0.16, *MSE* = 2.43, *p* = 0.69, η^2^ < 0.01, or delay, *F*(2, 246) = 0.80, *MSE* = 2.43, *p* = 0.45, η^2^ = 0.01, or interaction between collaboration and delay, *F*(2, 246) = 1.40, *MSE* = 2.43, *p* = 0.25, η^2^ = 0.01. Again, we conducted a Bayesian analysis and found BF_10_ = 0.24, indicating that the null model (including main effects) is 4.2 times more likely than the interaction effect model, and this is moderate evidence for the null hypothesis. The error rates are very low overall, so these results must be considered with caution. Nonetheless, they suggest that the error correction present on the initial test did not persist on subsequent tests (see Table 6).

**Table 6:** Mean number (standard deviation) of extralist intrusions recalled on prose Test 2 as a function of retrieval condition and delay (*N* = 252)

	5 min	48 h	1 week
Prose passages			
Prior individual	1.43 (1.93)	1.29 (1.37)	1.95 (1.62)
Prior collaborative	1.45 (1.27)	1.55 (1.69)	1.43 (1.38)

## Discussion

The current experiment systematically examined the time course of collaborative inhibition and post collaborative benefits across word lists and prose passages. For correct recall, we obtained collaborative inhibition on initial tests of both word lists and prose passages, and post collaborative benefits that lasted 1 week. For false recall, we obtained error correction on initial tests of word lists and prose passages, and importantly, there were no lasting effects of error correction on subsequent tests. The results demonstrate that post collaborative benefits are long lasting and selective to veridical recall.

The current experiment demonstrated that post collaborative benefits are present after just 5 min, and they persist at 1 week. These findings are consistent Blumen and Stern (2011), who demonstrated that post collaborative benefits for unrelated words persist 1 week after multiple tests. These results are also somewhat consistent with Yaron-Antar and Nachson (2006). Although they found no difference in the number of items produced following a 1-week delay, they did find that those who collaborated to remember the prime minister’s assassination had a higher accuracy rate. Importantly, however, the current results extend these findings to more typical experimental procedures in collaborative memory research; in the current study, we used just one collaborative recall of word lists and prose passages. Given the powerful effect of repeated tests on organization and memory (e.g., Congleton & Rajaram, 2011), and the role of emotion in memory (cf. Wessel et al., 2015), it is notable and important that post collaborative benefits in the current study persisted following a single collaborative recall session of basic laboratory stimuli.

Also important is that in the current study, long-lasting post collaborative benefits generalized across both word lists and prose passages. Marion and Thorley (2016) demonstrated that, at least on immediate and short delayed tests, post collaborative benefits are least likely for materials with high interitem association. In the current study, the categorized word lists and prose passages were both somewhat interrelated (at least relatively more so than unrelated word lists), and yet we found post collaborative benefits for both. Most likely, this is because of the difference in the time course of post collaborative benefits. Marion and Thorley examined post collaborative benefits on immediate and short delay test (0–10 min), whereas the current study examined post collaborative benefits after relatively long delays including 48 h and 1 week. To our knowledge, this is the first study to demonstrate long-lasting post collaborative benefits across different materials.

Theoretically, the veridical recall results are consistent with reexposure effects (Rajaram & Pereira-Pasarin, 2010). Reexposure provides individuals with a second studying opportunity because they are exposed to the information first during study and again during collaboration (e.g., Rajaram, 2018; Rajaram & Pereira-Pasarin, 2010). Our results are consistent with reexposure because post collaborative benefits were detected after both a short delay and a long delay. In contrast, if post collaborative benefits were the result of desirable difficulty, post collaborative benefits should have been detected only after a long delay. We tested the idea that trying to maintain to-be-recalled items in the face of distraction and interference might strengthen the items and/or cause more effortful retrieval during collaboration. However, we found no evidence that collaboration is a desirable difficulty because the post collaborative benefits were evident after just 5 min. This suggests that the types of difficulties present during collaboration (e.g., interruptions, disruptions, others’ errors) may operate on a different time course than the types of difficulties present during individual testing. This is important in understanding the mechanisms underlying collaborative inhibition, desirable difficulties, and post collaborative benefits.

Interestingly, the patterns of collaborative inhibition and post collaborative benefits obtained for correct recall did not generalize to false items. Regarding the time course of false memory, we found a different pattern. Specifically, on the initial test, we found evidence of error correction, but there were no lasting effects of error correction on the subsequent tests of word lists or prose passages. This finding is consistent with previous literature demonstrating that the effects of error correction are short lived (e.g., Hood et al., 2023; Whillock et al., 2020). However, our findings are inconsistent with previous papers showing that collaboration can increase errors (e.g., Meade & Roediger, 2009) and that error correction persists (e.g., Rossi-Arnaud et al., 2020). Possible explanations for the different results include the collaborative instructions (i.e., turn-taking vs. free-for-all recall; Thorley & Dewhurst, 2007) and the different mechanisms underlying false memory across materials (i.e., associative, categorical, schematic, etc.; cf. Roediger, 1996). For example, turn-taking collaboration has been shown to produce higher levels of subsequent false recall in both the misinformation paradigm (e.g., Saraiva & Garrido, 2024), and the Deese–Roediger–McDermott (DRM) associative memory paradigm (e.g., Thorley & Dewhurst, 2007); however, the evidence is more mixed with the DRM paradigm (e.g., Maswood et al., 2022). Differences across experiments can also be explained by the different delays between collaboration and subsequent recall. Again, the novel contribution of the current experiment is to examine the time course of post collaborative false recall. To our knowledge, the current experiment is the first paper to examine false recall following collaboration across different materials and delays, and we found no evidence that error correction persists.

Theoretically, error correction occurs because individuals are less likely to generate false items when they are in a group and/or they are just as likely to generate the false items, but their partners can correct them (Ross et al., 2008). The results of the current study are consistent with these mechanisms. More interesting is how to explain the findings that this error correction did not persist. One possibility is source monitoring errors; on the subsequent test, participants could have remembered the errors, but not whether they said them during recall and/or if the item was corrected or not (cf. Johnson et al., 1993). It is also possible that the participants went along with their partner’s error correction but privately continued to believe the false memory and so reported it on the later test and/or simply reverted to their original errors on the subsequent test. Another explanation is that when recalling in groups, individuals may not suggest items with low confidence (or set a stricter criterion) to avoid making mistakes, but when recalling individually, they tend to report all items they remember, including those they are not confident about, to perform well on the test. Finally, it is possible that collaborative groups may incorporate and respond to accurate and false suggestions differently, and this may influence how likely the items are incorporated in the group and subsequent individual memory (Ekeocha & Brennan, 2008; Meade, 2013).

There are several limitations to the current study that highlight the need for future research. One limitation is the materials used. Although we did examine generalizability across word lists and prose passages, future work is necessary to examine unrelated word lists, emotional events, and other materials to better understand how and when post collaborative benefits might differ across materials and across the time course. Also, in the current study, participants always recalled word lists first and then prose passages, and they completed both recall sessions with the same collaborative partner. Future research can examine how recalling one type of material might influence recall of other materials, and if there are practice effects with recall and/or collaborative partners (cf. Choi et al., 2014; Greeley et al., 2024). Another limitation is the low level of false memory for the prose passages. Again, future research should directly compare the time course of error correction and post collaborative errors across a range of materials. The current study relied on categorized word lists, and future extensions to associated memory illusions, misinformation effects, or conjunction errors could help isolate mechanisms related to error correction on immediate and delayed tests. Finally, the current experiment considers 48-h and 1-week delays as measuring long-term effects of collaboration. This is just a starting point to begin to understand the lasting consequences of collaboration and how durable such effects are across materials, delays, partner characteristics, larger groups, and longer delays.

In summary, the current experiment was the first to examine the time course of collaborative inhibition and post collaborative benefits for word lists and prose passages. We found post collaborative benefits for veridical recall that lasted up to 1 week. However, we found no lasting effects of error correction, suggesting a different time course of post collaborative effects for veridical and false recall. These results inform our understanding of the lasting effects of collaboration on individual memory. We frequently recall with others and collaborate in daily life, and these results can begin to help us understand beyond the collaboration itself how the experience continues to shape and influence our memories.

## Data Availability

The datasets generated during and/or analyzed during the current study are available in the OSF repository (10.17605/OSF.IO/SV8CW).

## Declarations

### Open practices statement

This was a one-experiment study, and hypotheses, experimental design, and expected data analysis were preregistered at Aspredicted (https://aspredicted.org/blind.php?x=2eb5ye). Datasets are available in the OSF repository (10.17605/OSF.IO/SV8CW).
